# Lead heavy metal toxicity induced changes on growth and antioxidative enzymes level in water hyacinths [*Eichhornia crassipes* (Mart.)]

**DOI:** 10.1186/s40529-014-0054-6

**Published:** 2014-07-24

**Authors:** Srinivasan Malar, Sahi Shivendra Vikram, Paulo JC Favas, Venkatachalam Perumal

**Affiliations:** 1 Department of Biotechnology, Plant Genetic Engineering and Molecular Biology Lab, Periyar University, Periyar Palkalai Nagar, Salem, 636 011 TN India; 2 Department of Biology, Western Kentucky University, Bowling Green, 42101 KY USA; 3 School of Life Sciences and the Environment, University of Trás-os-Montes e Alto Douro, UTAD, Quinta de Prados, Vila Real, 5000-801 Portugal

**Keywords:** Heavy metal stress, Aquatic plant, Reactive oxygen species, Lead hyperaccumulation, Phytoremediation

## Abstract

**Background:**

Lead (Pb) heavy metal pollution in water bodies is one of the serious problems across the world. This study was designed to find out the effect of Pb toxicity on physiological and biochemical changes in *Eichhornia crassipes* (water hyacinth) seedlings.

**Results:**

The plant growth was significantly inhibited (50%) at 1000 mg/L Pb concentration. Accumulation of Pb was higher in root than in shoot tissues. The maximum level of Pb accumulation was noticed in roots (5.45%) followed by petiole (2.72%) and leaf tissues (0.66%). Increasing the Pb concentration gradually decreased the chlorophyll content. Intracellular distribution of Pb was also studied using SEM-EDX, where the Pb deposition was observed in both root and leaf tissues. MDA content increased in both the leaf and root tissues up to the 400 mg/L Pb treatment and slightly decreased at higher concentrations. The activity of antioxidative enzymes, such as APX and POX, positively correlated with Pb treatment, while in the case of SOD and CAT enzymes increased up to 800 mg/L treatment and then slightly decreased at higher concentration in both leaf and root tissues.

**Conclusions:**

These results suggest that water hyacinth plants have efficient mechanism to tolerate Pb toxicity, as evidenced by an increased level of antioxidative enzymes. Results clearly indicate that water hyacinth is a feasible plant for hyperaccumulation of heavy metals from polluted wetlands.

**Electronic supplementary material:**

The online version of this article (doi:10.1186/s40529-014-0054-6) contains supplementary material, which is available to authorized users.

## Background

Heavy metal pollution has become one of the important environmental problems worldwide. Metal pollutants are particularly difficult to remediate from the soil, water and air because, unlike organic pollutants that can be degraded to harmless small molecules, toxic elements, such as lead, mercury, cadmium, copper and zinc, are immutable by biochemical reactions. Phytoremediation technology has recently started to gain great importance for the removal of elemental pollutants from soil and water. Hyperaccumulator plants represent a resource for phytoremediation of metal polluted sites, as they can tolerate, uptake and translocate high levels of certain heavy metals that would be toxic to most organisms (Meagher and Heaton [2005]).

Among heavy metals, lead (Pb) is one of the most hazardous pollutants of the environment and Pb pollution in air, water and agricultural soil is an ecological concern due to its impact on human health and environment. The main sources of Pb pollution in the environment are mining and smelting of Pb ore, industrial effluents, fertilizers, pesticides, and municipal sewage sludge (Sharma and Dubey [2005]). It has adverse effects on both plant and animals. In plants, lead affects several metabolic activities in different cell components. Lead toxicity leads to decreases in the percentage of seed germination, as well as growth, dry biomass of roots and shoots, disruption of mineral nutrition (Sharma and Dubey [2005]), reduction in cell division and inhibition of photosynthesis (Ekmekci et al. [2009]).

In addition, Pb is reported to produce reactive oxygen species (ROS) and enhance antioxidant enzyme activity in plants (Mishra et al. [2007]). The ROS produced as a result of oxidative stress causes a variety of harmful effects in plant cells, such as inhibition of photosynthetic activity, inhibition of ATP production, lipid peroxidation, and DNA damage (Ruley [2004]). One of the major consequences is the enhanced production of reactive oxygen species (ROS), which damage cell membranes, nucleic acids and chloroplast pigments (Tewari et al. [2002]). A number of different reactive oxygen species, including the superoxide anion (O_2_^-.^), singlet oxygen (^1^O_2_), hydroxyl radical (·OH) and the hydrogen peroxide (H_2_O_2_) are produced when plants are under heavy metal stress, but these ROS can pose a severe threat when produced in larger amounts. Production of excess ROS in heavy metal stressed plants may be a consequence of the distribution of the balance between their production and the antioxidative enzyme activity, composed of enzymic antioxidants, such as superoxide dismutase (SOD), catalase (CAT), ascorbate peroxidase (APX) and peroxidase (POX) (Zhang et al. [2007b]; Jiang et al. [2010]). The superoxide radical (O_2_^.-.^), is scavenged in stressed plants by superoxide dismutase, which converts O_2_^-.^ to hydrogen peroxide (H_2_O_2_) (Reddy et al. [2005]). H_2_O_2_ is scavenged directly by catalase, converting it to H_2_O and O_2_. Peroxidases, such as ascorbate peroxidase and peroxidase, also scavenge H_2_O_2_ indirectly by combining it with antioxidant compounds such as ascorbate (Yingli et al. [2011]). The effect of Pb stress has been studied recently in various plant species, including *Sesbania drummondii* (Venkatachalam et al. [2007]); *Triticum aestivum* L. (Ekmekci et al. [2009]); *Sesuvium portulacastrum* and *Brassica juncea* (Zaier et al. [2010]); *Salsola passerine* and *Chenpodium album* L. (Hu et al. [2012]); and, in aquatic plants (Zhang et al. [2007a]; Piotrowska et al. [2009]; Singh et al. [2010]). Plants have divergent mechanisms for modulating heavy metal levels to adapt to a change in the concentration of metals in the polluted environment. Heavy metals are toxic to plants if their accumulation levels exceed the detoxification capacity of the plant tissues (Zhang et al. [2007b]).

Aquatic ecosystems are more sensitive to heavy metal pollutants than terrestrial ones. Large quantities of municipal wastes and industrial effluents are being released to the aquatic environment in major cities where the environment is highly polluted by various hazardous chemicals and metals. Therefore, there is an urgent need for a low cost method to remediate the polluted sites. In order to remove the pollutants using phytoremeidation technology, it is essential to identify a suitable aquatic plant species with high biomass. *E. crassipes* (Water hyacinth) a free floating plant present in aquatic ecosystems, such as ditches, ponds and lakes, was selected for this study. Water hyacinth can be easily cultivated and could produce high biomass in aquatic ecosystems. Though this species has the capacity to accumulate various heavy metals, there is insufficient information about the plant’s response to Pb exposure under hydroponic systems (Mishra et al. [2007]; Odjegba and Fasidi [2007]; Zhang et al. [2007b]; Mahamadi and Nharingo [2010]; Chunkao et al. [2012]; Singh and Kalamdhad [2013]). In this study, we hypothesize that, due to the multi tolerance mechanism, *E. crassipes* has the potential to deal with stress at the cellular level and we examined its capability to cope with oxidative stress caused by Pb ions at low, medium and high concentrations in the hydroponic solution. When *E. crassipes* plants were exposed to different doses of Pb ions and growth rate, biomass, photosynthetic apparatus and antioxidant enzymes, SOD, APX, CAT, and POX were determined. The results obtained in the present study could be useful for understanding the role of *E. crassipes* antioxidative defense system in efficient Pb tolerance and detoxification strategy adopted by the plants.

## Methods

### Plant material and growth conditions

Water hyacinths seedlings were used for this study. The plants were washed thoroughly with tap water followed by de-ionized water. The plants were grown in plastic cups containing Hoagland nutrient solution (500 mL) (Hoagland and Arnon [1950]) with continuous aeration. The pH of the nutrient solution was 5.8. The seedlings were grown in hydroponics under green house condition for 7 days. In order to select the lead concentrations for treatment, various doses of Pb (NO_3_)_2_ (0, 100, 200, 300, 400, 500, 600, 700, 800, 900, 1,000 and 1,500 mg/L) were tried in the preliminary screening of the water hyacinths seedlings. Based on the lead toxicity symptoms and physiological growth of the seedlings, the following doses were finally selected and lead treatment was performed for 10 days. Seedlings were treated with different concentrations of Pb (NO_3_)_2_ (100, 200, 400, 600, 800, 1000 mg/L), while seedlings without Pb treatment were used as a control. For each treatment, triplicates were maintained. After ten days of treatment, the seedlings were thoroughly washed with EDTA and root, petiole and leaf samples were used for Pb content analysis. In addition, tolerance index, photosynthetic pigments, MDA content and anti-oxidative enzyme activities of the seedlings were also measured. Scanning Electron Microscopic - EDX observations were also performed to determine the localization and translocation path of Pb in plant tissues.

### Determination of seedlings growth tolerance index (GTI)

The length of the longest root and shoot of each plant was recorded after 10 days of treatment period. Root and shoot length of seedlings were measured and growth inhibitory rate (%), of root and shoot were calculated according (Wilkins [1978]):

1 $$\;\mathrm{Growth}\;\mathrm{Tolerance}\;\mathrm{Index}\;\%=\frac{\mathrm{Growth}\;\mathrm{in}\;\mathrm{solution}\;+\;\mathrm{metal}}{\mathrm{Growth}\;\mathrm{in}\;\mathrm{solution}\;-\;\mathrm{metal}}\times 100$$

### Estimation of seedling biomass and relative water content (RWC)

For biomass and RWC analysis, plants were separated into shoots and roots. Wet plant biomass (FW) was immediately determined. The samples were dried in a hot air oven for 48 hours at 65°C for determination of dry weight biomass (DW). The relative water content (RWC) was also calculated as described by Chen et al. ([2009]).

2 $$\mathrm{RWC}\left(\%\right)=\left[\left(\mathrm{FW}–\mathrm{DW}\right)/\mathrm{FW}\right]*100$$

### Lead content analysis and translocation factor

Lead treated plant tissues (shoots and roots) were dried in a hot air oven at 65°C for 48 hours to remove all the moisture content. The oven dried tissues were ground into fine powder and used for metal analysis by ICP-MS (Department of Biology, Western Kentucky University, Bowling Green, KY, USA). The level of lead content in root and shoot tissues was quantified according to the method of Israr et al. ([2006]). Translocation factor (TF) was calculated according to the method of Marchiol et al. ([2004]).

3 TF=MetalConcentrationinshoots/MetalConcentrationinroots

### Estimation of photosynthetic pigment contents

Photosynthetic pigments were extracted from leaves using 80% (v/v) acetone and chlorophyll *a*, *b*. Carotenoids content was determined spectrophotometrically at 665, 649 and 470 nm according to (Lichtenthaler [1987]) and expressed in mg/g FW.

### Scanning electron microscopy and energy dispersive X-ray spectroscopy

In order to determine the cellular localization of lead, 1000 mg/L of lead treated plant tissues (leaves and roots) were freeze- dried overnight at – 30°C under a vacuum (15–25 torr) using a Labconco’ freeze-dry system (Freezone 4.5, Labconco , Kansas city, MO, USA). Samples were mounted on aluminum stubs with double-sided carbon tape. The SEM observations were performed using a JEOL JSM-5400 LV scanning electron microscope in a low vacuum mode using backscattered electron imaging.

### Measurement of MDA contents

Lipid peroxidation was estimated by measuring the total amount of malondialdehyde (MDA) contents, as described by Davenport et al. ([2003]). Briefly, fresh leaf and root tissues (0.2 g) were homogenized using 2 ml of 5% (w/v) trichloroacetic acid in an ice bath and centrifuged at 10,000 rpm for 10 minutes at 4**°** C. About 2 ml supernatant was mixed with 2 ml of 0.67% (w/v) thiobarbituric acid and the mixture was incubated in a boiling water bath for 30 minutes, then cooled and centrifuged. The absorption of supernatant was carried out at 450, 532 and 600 nm. The MDA content was calculated as described below:

4 $$\mathrm{MDA}\left(µ\mathrm{mol}\;g^{-1}\right)=\left[6.45\;\times \left(A_{532}-A_{600}\right)-\left(0.56\;\times A_{450}\right)\right]\times \mathrm{Vt}/W$$

5 WhereVt=0.0021;W=0.2g

### Determination of antioxidative enzyme activities

Both root and leaf tissues (>200 mg) from water hyacinth seedlings were homogenized separately in a pre-chilled mortar and pestle under ice-cold conditions with 2.0 ml of extraction buffer [50 mM phosphate buffer (pH 7.5), 0.5 mM ascorbate and 1 mM EDTA]. The homogenate was centrifuged at 10,000 rpm for 15 minutes. The supernatant was used for the measurement of SOD, CAT, APX and POX antioxidative enzyme activities. In addition, the protein content was also measured according to the method of Bradford ([1976]), using bovine serum albumin (BSA) as standard.

The SOD activity was quantified by measuring its ability to inhibit the photochemical reduction of nitroblue tetrazolium (NBT) (Beauchamp and Fridovich, [1971]). The reaction mixture (3 ml) contained 100 mM potassium phosphate buffer (pH 7.8), 0.1 mM EDTA, 13 mM methionine, 2.25 mM NBT, 60 μM riboflavin and enzyme extract. After mixing, the contents in the cuvette were illuminated (40 watts light) for 15 minutes. Enzyme extract kept in the dark served as blank, while buffer with no enzyme extract kept in the light served as control. The absorbance was measured at 560 nm against a blank using a UV–visible spectrophotometer. NBT reduction in the light was measured in the presence and absence of enzyme extract. SOD activity was calculated as absorbance of control minus absorbance of sample, giving the total inhibition. One unit of activity was the amount of enzyme required for 50% reduction in color and was expressed in units of the enzyme (mg/protein/h).

Catalase activity was determined by measuring the decomposition of hydrogen peroxide. About 100 μl of enzyme extract was added into the reaction mixture containing 50 mM phosphate buffer (pH 7.0) and 20 mM H_2_O_2_. The decrease of the absorbance at 240 nm was recorded. Activity was calculated using an extinction coefficient of 39.04 mM^−1^ cm^−1^. One unit of CAT activity was defined as the amount required for decomposing 1 μmol of hydrogen peroxide/min/mg protein under assay conditions (Beer and Sizer [1952]).

APX activity was measured according to the method of Nakano and Asada ([1987]). The reaction mixture (3 ml) contained 100 mM potassium phosphate buffer (pH 7.0), 0.5 mM ascorbate, 0.3 mM H_2_O_2_ and enzyme extract. The oxidation of ascorbic acid was measured by the decrease in absorbance at 290 nm for 3 min using a UV–visible spectrophotometer. The enzyme activity was calculated using the extinction coefficient 2.8 mM^−1^ cm^−1^ and expressed in units/mg protein. One unit of enzyme was the amount necessary to decompose 1 μmol of substrate/min at 25 ºC.

POX activity was estimated according to the method of Zhang et al. ([1995]). The reaction mixture (3 ml) contained 100 mM potassium phosphate buffer (pH 6.1), 96 mM guaiacol, 12 mM H_2_O_2_ and enzyme extract. The oxidation of guaiacol was measured by the increase in absorbance at 470 nm. The enzyme activity was calculated using the extinction coefficient 25.5 mM^−1^ cm^−1^ and expressed in units (mg/protein). One unit of enzyme was the amount necessary to decompose 1 μmol of substrate per min.

### Statistical analysis

Each experiment was performed in triplicate and data was recorded. Statistical analysis was performed using Graphpad InStat Software. Significant differences among treatments were analyzed by one-way ANOVA, using p ≤ 0.05 as a significant level and Tukey-Kramer multiple comparisons tests conducted for pair wise comparisons between treatments.

## Results

### Effect of Pb concentration on seedlings growth, biomass and relative water content

Growth inhibition is a common response to heavy metal stress and is also one of the most important agricultural indices of heavy metal tolerance. Lead is not generally considered to be an essential element for plant growth. The effect of Pb on seedling growth seems to be different with regards to plant species, cultivars, organs and metabolic processes (Sharma and Dubey [2005]). Water hyacinth seedlings grown in different concentrations of Pb (NO_3_)_2_ exhibited inhibition of both root and shoot growth, with shoots being affected more than roots. After 10 days of Pb treatment, the reduction of seedling length was 56% and 44% in root and shoot respectively (Table 1). Plants do not show any visible toxicity symptoms up to 800 mg/L Pb treatment. However, Pb treatment at 1000 mg concentration showed toxicity symptoms like chlorosis and drying at edges in seedlings. This may be due to heavy metal toxicity and accumulation of Pb content in their leaves. Water hyacinth seedlings showed 33.28% biomass reduction at 1000 mg/L Pb exposure when compared to the control. Relative water content (RWC) in water hyacinth increased slightly, up to 400 mg/L Pb concentration, and decreased slightly at higher concentrations compared to the control (Table 2).

**Table 1:** **Effects of lead heavy metal exposure on growth parameters of** ***E. crassipes*** **seedlings**

Pb Conc. (mg/L)	Root length (cm)	Shoot length (cm)	Growth rate inhibition (GRI)%	
			Root	Shoot
0.0	22.33 ± 0.81^a^*	23.83 ± 2.23^a^*	0.0	0.0
100	20.41 ± 1.67^b^	20.25 ± 0.83^b^	8.6	15.03
200	20.16 ± 0.76^b^	19.50 ± 0.56^b^	9.72	18.18
400	18.41 ± 0.91^c^	17.75 ± 1.38^c^	17.56	25.52
600	15.75 ± 1.26^d^	17.00 ± 0.76^c^	29.38	28.67
800	13.25 ± 1.25^d^	16.66 ± 1.08^d^	40.67	30.09
1000	09.83 ± 0.79^e^	13.25 ± 0.99^e^	55.98	44.34

*Values are expressed as mean ± SE (n = 3), different letters in each column indicate significant difference at p < 0.05 levels.

**Table 2:** **Effects of lead heavy metal on plant biomass and relative water content** (**RWC**) **of** ***E. crassipes*** **seedlings**

Pb Conc. (mg/L)	Biomass (g/seedlings)		Relative water content (RWC)%
	Fresh weight (FW)	Dry weight (DW)	
0.0	10.03 ± 0.033^a^*	0.727 ± 0.004^a^*	92.73 ± 0.06^e^*
100	09.95 ± 0.043^a^	0.712 ± 0.007a	92.84 ± 0.07^d^
200	09.63 ± 0.031^b^	0.640 ± 0.003^b^	93.35 ± 0.05^b^
400	08.84 ± 0.141^c^	0.564 ± 0.012^b^	93.61 ± 0.04^a^
600	07.95 ± 0.142^d^	0.532 ± 0.005^c^	93.30 ± 0.03^b^
800	07.26 ± 0.167^d^	0.500 ± 0.006^c^	93.11 ± 0.03^c^
1000	06.90 ± 0.063^e^	0.485 ± 0.001^d^	92.97 ± 0.04^d^

*Values are expressed as mean ± SE (n = 3), different letters in each column indicate significant difference at p < 0.05 levels.

### Lead accumulation in water hyacinth seedlings

**Figure 1:**
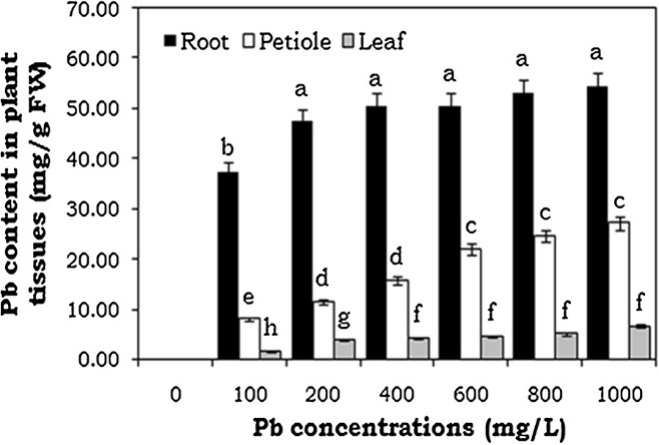
**Accumulation of lead heavy metal content in root, petiole and leaf tissues of** ***E*** **.** ***crassipes*** **seedlings.** The error bars indicate mean ± SE (n = 3), followed by different letter are statistically significant at p ≤ 0.05 levels.

Accumulation of Pb content in water hyacinth seedlings was dependent on the concentration present in the growth medium. The Pb accumulation level was found to be higher in roots followed by petiole and leaf tissues. The level of Pb accumulation in roots, petioles and leaves showed positive linear relationships with the Pb concentration in the nutrient solution. The maximum accumulation of Pb content was 5.45% in roots followed by petioles (2.70%) and leaf tissues (66%) (Figure 1). The translocation factor value was found to be less than 1. Though, Pb was largely stored in plant roots exposed to 1000 mg/L Pb treatment, only lesser amounts of Pb were translocated to aerial parts of the plants. This result clearly indicate that the large amount of metal content was accumulated in roots, but only lower levels of Pb content were translocated into shoots of water hyacinth seedlings.

### Effect of Pb on photosynthetic pigment contents

**Figure 2:**
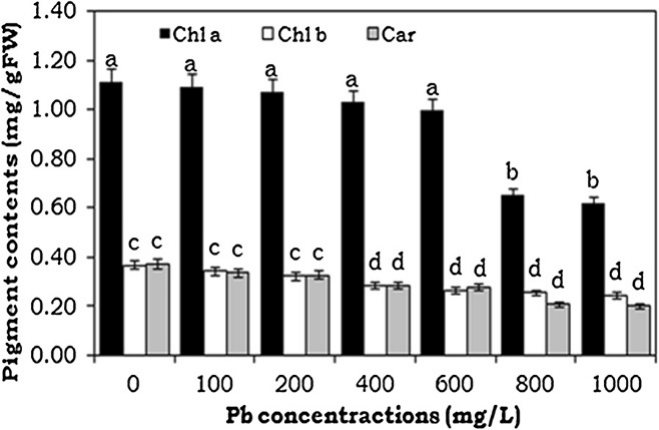
**Effects of lead heavy metal exposure on chlorophyll contents (Chl** ***a*** **,** ***b*** **and car) in** ***E*** **.** ***crassipes*** **seedlings.** The error bars indicate mean ± SE (n = 3), followed by different letter are statistically significant at p ≤ 0.05 levels. Chl *a*-Chlorophyll *a*, Chl *b* -Chlorophyll *b*, Car-Carotenionds.

The effect of different concentrations of lead treatment (100 to 1000 mg/L) on photosynthetic pigments is depicted in Figure 2. Photosynthetic pigment contents of leaves were decreased by increasing the Pb level in the growth medium. The reduction of chlorophyll *a*, *b* and carotenoid contents was 55%, 67% and 55%, respectively, in 1000 mg/L Pb treated plants compared to the control.

### Scanning electron microscopic observations

**Figure 3:**
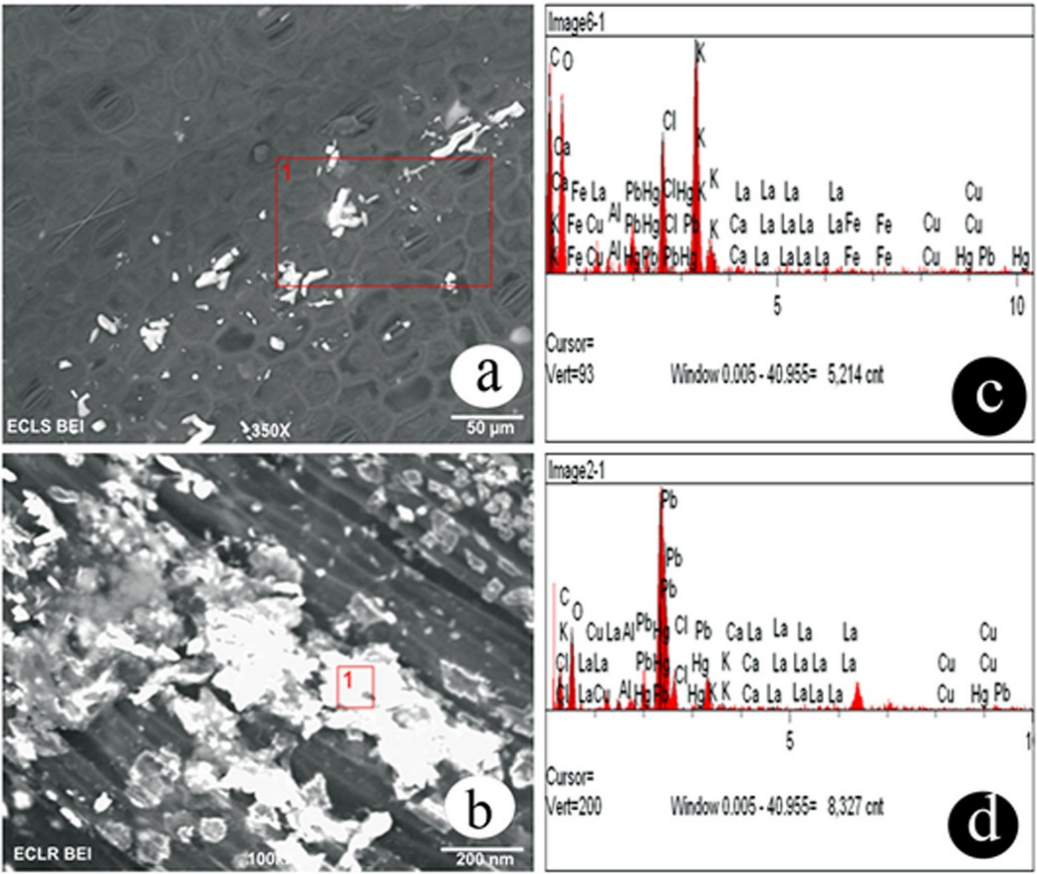
**SEM micrograph and EDAX spectra of lead distribution in leaf (a, c) and root (b, d) tissues of** ***E*** **.** ***crassipes*** **grown in hydroponic solution with 1000 mg/L Pb concentration.**

Scanning Electron Microscopy equipped with Energy Dispersive X-ray Spectrometer (EDX) analysis was performed to determine the location and transport of Pb ions in water hyacinth root and leaf tissues grown at 1000 mg/L Pb concentration. It was observed that most of the Pb metal ion had accumulated in the roots. A distinct signal and high atomic values for Pb were also noticed in Energy dispersive X-ray (EDX) analysis. Lead could be chelated to organic compounds, as the EDX spectrum of peaks (Figure 3).

### Effect of Pb stress on MDA content

**Figure 4:**
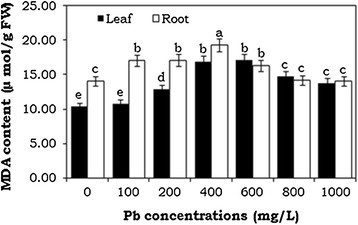
**Effects of lead heavy metal exposure on MDA content (μ mol/g FW) in leaf and root tissues of** ***E*** **.** ***crassipes*** **seedlings.** The error bars indicate mean ± SE (n = 3), followed by different letter are statistically significant at p ≤ 0.05 levels.

The level of lipid peroxidation in water hyacinth seedlings was estimated by MDA content (Figure 4). The total MDA content was increased with the increasing lead concentration (up to 400 mg/L) and slightly decreased at higher Pb doses. Maximum MDA content was 15% and 37% in leaf and root tissues respectively, compared to the control.

### Effect of Pb exposure on antioxidative enzyme activity

**Figure 5:**
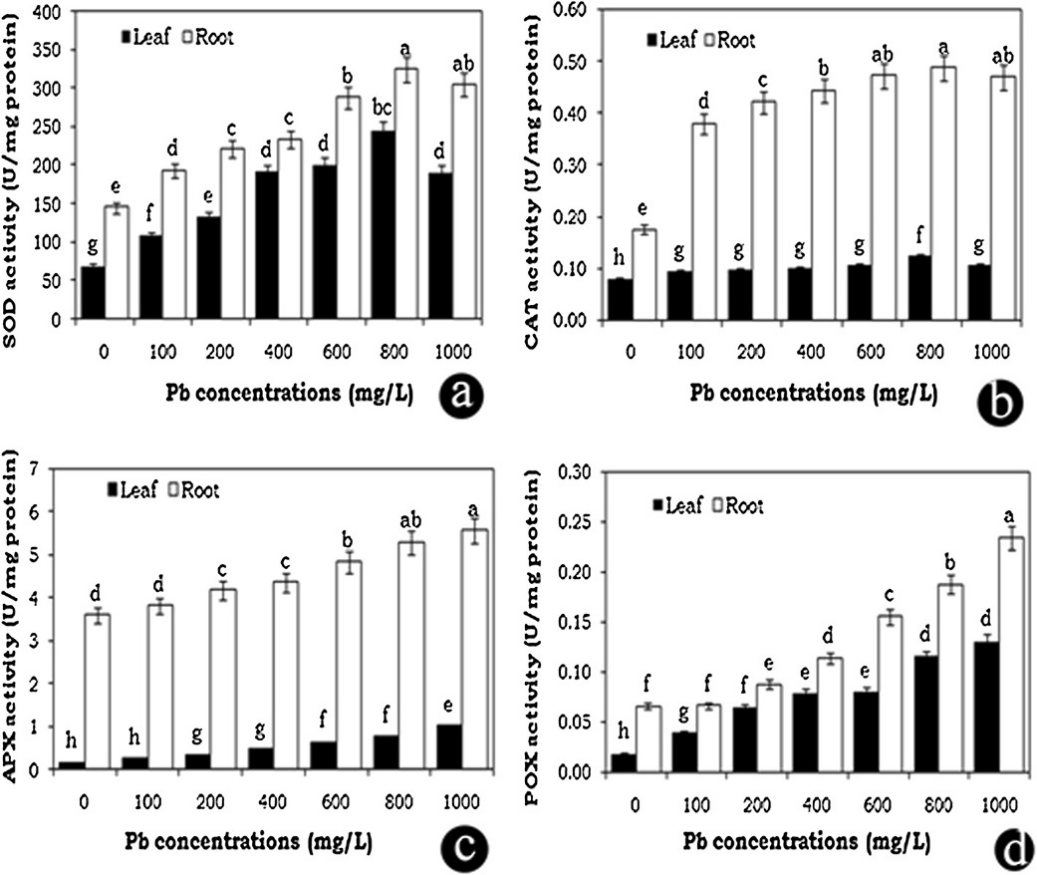
**Effects of lead heavy metal stress on SOD (a), CAT (b), APX (c) and POX (d), antioxidative enzyme activities in leaf and root tissues of** ***E*** **.** ***crassipes*** **seedlings.** The error bars indicate mean ± SE (n = 3), followed by different letter are statistically significant at p ≤ 0.05 levels.

The relation between superoxide dismutase activity and the concentration of Pb in the growth medium is depicted in Figure 5a. In water hyacinth seedlings, SOD activity was increased significantly in a concentration dependent manner, up to 800 mg/L Pb in both leaf and root tissues. Maximum SOD activity was 251% and 123% higher in leaf and root tissues of Pb doses respectively, compared to the control. However, the SOD activity was slightly decreased at 1000 mg/L Pb concentration. There was a positive correlation between the SOD activity and the Pb dose.

In the present study, the catalase activity showed increases in leaf and root tissues up to 800 mg/L Pb concentration (60% and 177% increment in leaf and root tissues). Thereafter it was slightly decreased at 1000 mg/L (Figure 5b). However, the catalase activity showed 35% and 166% increases even at 1000 mg/L Pb treatment of leaf and root tissues, compared to the control. In general, the catalase activity was significantly increased at increasing concentrations of Pb treatment.

The APX activity in leaf and root tissues was increased significantly with increasing concentrations of Pb up to 1000 mg/L (Figure 5c). APX activity also showed 537% and 55% increases in leaf and root tissues respectively, compared to the control. The present result clearly shows that APX activity was positively correlated with Pb treatment to maintain the balance between the formation of ROS and their removal in water hyacinth seedlings.

The application of different Pb concentrations had significant effects on peroxidase activity in water hyacinth seedlings. The peroxidase activity was increased with increasing the Pb concentration up to 1000 mg/L in both leaf and root tissues (Figure 5d). Compared to the control, POX activity was increased about 589% and 254% in leaf and root tissues, respectively. Data on peroxidase activity was statistically significant and positively correlated with Pb treatment. It was observed that the water hyacinth plant has the ability to maintain high levels of POX activity at higher concentrations of Pb heavy metal treatment.

## Discussion

In this study, the major response of plants to Pb toxicity is the inhibition of plant growth and induction of oxidative stress in plants. There were strong morphological differences between lead-treated and control plants. The absence of visual damage to the seedlings suggests that water hyacinth plants have efficient mechanisms to tolerate Pb metal induced stress under the present experimental conditions. The most noticeable symptoms of Pb toxicity in water hyacinths were the inhibition of plant growth. In addition, plant biomass is a good indicator for characterizing the growth performance of plants in the presence of heavy metal. In the present study, as the lead treatment was reduced, water hyacinth plant growth rates and overall biomass production decreased. Similar response to lead treatment was previously noticed in various plants (Piechalak et al. [2002], [2003]; Brunet et al. [2008]). Decreased plant growth might be associated with the inhibition of mitotic index noticed with Pb and Cd heavy metal treatment (Vecchia et al. [2005]). Relative water content (RWC) change has been suggested as an indicator of phytotoxicity after heavy metal stress (Zn and Cr) in Indian mustard and Chinese brake fern (Su et al. [2005]). Relative water content in leaves was slightly higher in lead treated plants than in the control at the end of the treatment. It is most likely that lead treatment induced stomatal closure, triggered over the course of the experiment due to the atmospheric carbon fixing activities that were compromised as a consequence (Brunet et al. [2008]).

Plant tolerance to heavy metal stress is estimated based on their root and/or shoot growth inhibition by the metal present in a nutrient solution (Michalal and Wierzbicka [1998]; Wang and Zhou [2005]). Earlier studies on the mechanism of Pb toxicity suggested that Pb binds to nucleic acids and causes aggregation and condensation of chromatin, as well as inhibiting the process of replication, transcription and ultimately affecting cell division and plant growth (Johnson [1998]). In addition, other symptom, such as chlorosis and drying of leaf edge has also been reported in *Sesbania drummondii* following Pb treatment (Venkatachalam et al. [2007]).

Accumulation of heavy metal content in different plant tissues was greatly varied depending on the Pb concentrations. In this study, accumulation of Pb content in roots of water hyacinth plants was significantly higher, followed by petiole and leaf tissues. Depending on the Pb treatment, the translocation of Pb from root to shoot was 38.27%. Roots were washed with EDTA prior to metal analysis. In order to differentiate between exchangeable and non-exchangeable Pb ions, desorption solutions, viz. EDTA and HETDA, have been used earlier on lead-treated root systems (Huang and Cunningham [1996]; Brunet et al. [2008]). It has been reported that short-term Pb exposure mostly consisted of exchangeable Pb ions, while long-term Pb treatment, as was examined in the present experiment, mostly involved non-exchangeable Pb. An efficient Pb accumulation mechanism in water hyacinth roots could represent a new and interesting phenomenon for establishment of phytoremediation strategies, in which higher levels of the contaminant remain tightly attached to plant tissues. Recent reports also show that Pb accumulation was found to be higher in the roots than in the shoots of *Brassica rapa* (Cenkci et al. [2010]). The ability of the water hyacinth plant in accumulation and tolerance to Pb ions indicates that this plant species may have an efficient hyperaccumulation mechanism for removal of Pb metal ions from contaminated sites and water bodies.

With the accumulation of Pb ions in water hyacinth plant tissues, we observed effects on the photosynthetic pigments and decreased levels of chlorophyll content in this study. Similarly, reductions in the level of photosynthetic pigments, including Chl- *a*, *b* and carotenoids, after exposure to heavy metals, including Pb, has been observed in many plant species (Mishra et al. [2007]; Piotrowska et al., [2009]; Singh et al. [2010]). It has also been reported that alterations in photosynthetic activity and the absorption and distribution of essential nutrients lead to reduced plant growth. The reduction of Chl *b* was more than overall Chl content. This can be associated with the alteration in pigment composition of photosynthetic approach that possesses lower level of light harvesting chlorophyll proteins (LHCPS) (Loggini et al. [1999]; Gill et al. [2012]). The decreased level of LHCPS is an adaptation defense mechanism of leaves and plants, helping them survive under adverse conditions. Photosynthesis in higher plants is more sensitive to heavy metal treatments, affecting biosynthesis of cholorphyll and accessory pigments (Mobin and Khan [2007]; Ahemad and Khan [2009]; Iqbal et al. [2010]; Gill et al. [2012]). It can be assumed that lead may inhibit chlorophyll biosynthesis by impairing the uptake of essential photosynthetic pigment elements, such as magnesium, potassium, calcium and ion (Piotrowska et al. [2009]). EDAX attachment with scanning electron microscopic analysis is known to provide information on the chemical analysis of the fields that are being investigated or the composition at specific locations. The results of SEM studies indicated that more important effect of Pb toxicity was decreasing the stomata ostiole and increasing the size of guard cells. Therefore Pb enters the leaf through stomata openings and their toxicity may disturb the physiological activity of plants (Sharma and Dubey, [2005]). In this study, EDAX with SEM analysis revealed the presence of lead on plant tissues (leaf and root) of 1000 mg/L Pb treatment.

A decreased rate of photosynthetic pigment accumulation in association with Pb treatment may be the consequence of peroxidation of chloroplast membranes due to increased level of ROS generation. This result is consistent with the enhanced level of H_2_O_2_ and peroxide production in water hyacinth plants treated with lead. The localization of Pb ions mainly observed in the root xylem suggests that it is the main pathway of Pb transport from root to shoot. Similar observations were also reported by Sharma et al. ([2004]).

The present study strongly suggests that Pb toxicity *in situ* leads to triggers of some of the key enzymes of the antioxidant defense system in water hyacinth plants. To resist oxidative damage, the antioxidant enzymes and certain metabolites, including MDA content present in plants, play a vital role leading to adaptation and the ultimate survival of plants under stress conditions (Zhang et al. [2007b]). MDA is the product of lipid peroxidation when plants are under stress, and it is often used as an indicator of the extent of oxidative stress (Chen et al. [2009]; Hu et al. [2012]). In the present study, MDA content in water hyacinth seedlings was increased significantly up to 400 mg/L of Pb treatment and then slightly decreased at higher concentrations. This is attributed to the activity of antioxidative enzymes to reduce H_2_O_2_ levels and therefore minimize the cell damage to membranes (Zhang et al. [2007b]). The present study suggests that Pb toxicity *in situ* triggers some of the key enzymes involved in antioxidant defense systems in water hyacinth seedlings.

An increased level of antioxidative enzymes involved in H_2_O_2_ detoxification, such as superoxide dismutase, catalase, ascorbate peroxidase, and peroxidase was observed in both leaf and root tissues of Pb treated plants, compared with the control. Superoxide dismutase is considered as a first defense system against ROS, as it acts on superoxide free radicals, which are produced in different compartments of the cell and are precursors of the other ROS (Alscher and Erturk [2002]). In general, the current results show an increased level of SOD activity in water hyacinth plants growing under lead treatment. However, the SOD activity was slightly decreased at higher concentrations of Pb (1000 mg/L), probably because of the harmful effects of over-production of H_2_O_2_ or its poisonous ROS derivatives. This may be because initially the SOD activity was increased as a result of the formation of ROS by Pb exposure. Similar results were reported by Zhang et al. ([2007a]); Piotrowska et al. ([2009]) and Feng-tao et al. ([2013]). Enhanced level of SOD activity may be attributed to the production of more active oxygen species (AOS) or over expression of genes encoding SOD. A slight decrease in SOD activity noticed at the higher Pb dose may be due to the inhibition of enzyme activity by excess H_2_O_2_ content that is a product in various cellular compartments (Mishra et al. [2006]). In many plant species, heavy metals have been reported to cause oxidative damage due to the production of excess ROS (Verma and Dubey [2003]). To resist oxidative damage, the antioxidant enzymes and certain metabolites present in plants play an important role leading to adaptation and ultimate survival of plants under stress conditions (Verma and Dubey [2003]). The enhancement of H_2_O_2_ content may be correlated with the increased level of SOD activity following Pb treatment. Therefore, the presence of excess of H_2_O_2_ content should be toxic and must be eliminated by conversion of H_2_O in subsequent reactions. Protecting the Pb stressed plants from the damaging effects of H_2_O_2_ requires the induction of different antioxidative enzymes. The activities of CAT, APX and POX were enhanced in water hyacinth plants following Pb exposure. Results showed that CAT activity was significantly increased up to 800 mg/L Pb treatment and slightly decreased at higher Pb concentrations. Catalase involved in the main defense mechanism against accumulation and toxicity of AOS, such as hydrogen peroxide, may play a key role in decreasing H_2_O_2_ content levels in plant cells. CAT enzymes eliminate H_2_O_2_ by breaking it down directly to form water and oxygen. It is likely that excess production of ROS by heavy metal stress can inactivate CAT activity at higher concentrations of heavy metals, probably by inactivating the enzyme-bound to heme group (Willekens et al. [1997]). Apparently, the decreased CAT activity at high Pb dose was compensated by the enhanced activity of two other H_2_O_2_ degrading antioxidative enzymes i.e., APX and POX. Contrary to our results, a decline in the activity of catalase with an increase heavy metal concentration has also been observed in *Lemna gibba* (Parlak and Yilmaz [2013]) and in *Becopa monnera* (Mishra et al. [2006]).

In the present study, enhanced APX activity in water hyacinth is generally correlated with an adaptive mechanism to increase levels of AOS content produced by Pb metal ions. Enzymes of ascorbate are localized mainly in chloroplasts and also in other cellular organelles and cytoplasm, where they play important role in combating oxidative stress. APX activity was increased significantly with increasing the concentration of Pb up to 1000 mg/L in leaf and root tissues. A positive correlation between APX activity and excess ROS may be attributed in effective scavenging of H_2_O_2_ content to protect stressed plants against oxidative damage induced under lead stress. Similar to the results observed in the present study, an increase in APX activity in plants following exposure of heavy metals was reported by Piotrowska et al. ([2009]) and Feng-tao et al. ([2013]).

Peroxidase is one of the principle enzymes involved in the elimination of active oxygen species (AOS) under stress. POX catalysis H_2_O_2_ is dependent oxidation of substrate. Enhanced POX activity levels have been shown to associate with plant adaptation to severe Pb heavy metal stress and the stimulation of POX activity is likely to be involved in excess of H_2_O_2_ content detoxification in water hyacinth plants to grow in the presence of 1000 mg/L Pb ions. Peroxidase has a higher affinity for H_2_O_2_ than CAT. Pb stress resulted in increased POX activity up to 1000 mg/L Pb treatment of water hyacinth plants. This also indicated that water hyacinth plants more efficiently avoided damaged from heavy metal lead stress. It is reported that POX could function as effective quenchers of reactive intermediary forms of oxygen and peroxy radicals, stimulated by increased heavy metal doses in plant cells (Radotic et al. [2000]). Peroxidase is widely distributed in the plant kingdom and is one of the principle enzymes involved in the elimination of active oxygen species (AOS). POX consumes H_2_O_2_ to generate phenoxy compounds that are polymerized to produce cell wall components, such as lignans (Hu et al. [2012]). The present results are in agreement with other reports showing the positive effects of heavy metal treatment on antioxidative defense systems (Hou et al. [2007]; Jin et al. [2008]; Piotrowska et al. [2009]).

At a higher concentration of Pb treatment (1000 mg/L), an increased level of antioxidative enzymes activities in both leaf and root tissues suggests that enhanced levels of ascorbate peroxidase and peroidase in water hyacinth plants upon exposure to Pb. The stimulation of SOD, CAT, POX and APX in response to Pb treatment indicates their role in ROS detoxification in water hyacinth seedlings. In this study, enhanced ascorbate peroxidase and peroxidase activity strongly support the hypothesis that the H_2_O_2_ content mobbing ascorbate peroxidase cycle is likely to be activated in water hyacinth treated with Pb heavy metals, especially at 1000 mg/L exposure. An increased activity of the antioxidative enzymes in water hyacinth plants in response to Pb exposure (100 to 1000 mg/L) is likely to protect plants from heavy metal induced oxidative stress by activating multi defense mechanisms and for better growth in polluted environments.

## Conclusion

In conclusion, an efficient adaptation to hydroponics and the valuable Pb accumulation observed for water hyacinth plants, especially at higher doses of heavy metal, shows the great potential of this plant species for the decontamination of pollutants in water-based systems. In this study, water hyacinth plants adapted to higher doses of Pb treatment and the level of antioxidative enzymes was enhanced significantly. Results strongly suggest that water hyacinths are not affected by oxidative stress, in spite of the presence of higher dose of Pb in the hydroponic medium, as would be anticipated for a species that has efficiently survived in a highly polluted environment. Therefore, the data obtained could be used to demonstrate how water hyacinths trigger antioxidant reactions upon exposure to Pb. Increased SOD, CAT, APX and POX activity appear to play key roles in the antioxidant defense response of water hyacinth seedlings when exposed to Pb heavy metal toxicity. These findings clearly show that enhanced antioxidant enzyme mechanisms in water hyacinth seedlings to heavy metal stress could help to overcome metal toxicity from ROS detoxification. Interestingly, water hyacinths, with the aid of its root and petiole, could serve as an important plant species in phytoremediation of Pb heavy metal polluted wetland areas where no other species with high biomass is able to grow.

## Authors’ original submitted files for images

Below are the links to the authors’ original submitted files for images. Authors’ original file for figure 1 Authors’ original file for figure 2 Authors’ original file for figure 3 Authors’ original file for figure 4 Authors’ original file for figure 5
