# Supplementary figures and images for: Lead heavy metal toxicity induced changes on growth and antioxidative enzymes level in water hyacinths [Eichhornia crassipes (Mart.)]
Source: Bot Stud. 2014 Jul 24;55:54. doi: 10.1186/s40529-014-0054-6 (PMC5430585; doi:10.1186/s40529-014-0054-6)

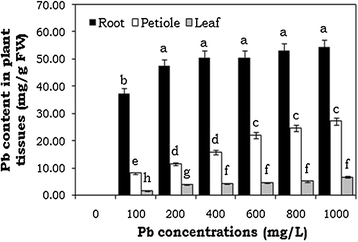

Supplement: Supplementary file 1 — Authors’ original file for figure 1 [file 40529_2014_9054_MOESM1_ESM.gif]

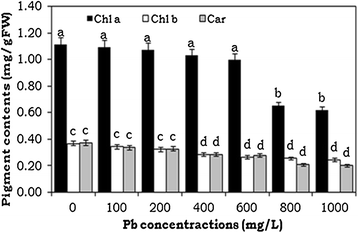

Supplement: Supplementary file 2 — Authors’ original file for figure 2 [file 40529_2014_9054_MOESM2_ESM.gif]

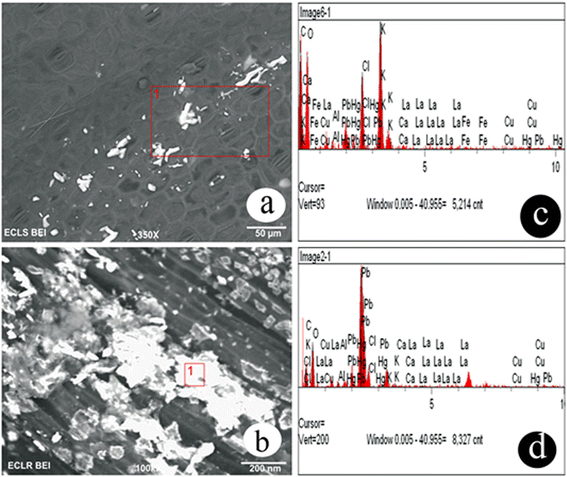

Supplement: Supplementary file 3 — Authors’ original file for figure 3 [file 40529_2014_9054_MOESM3_ESM.gif]

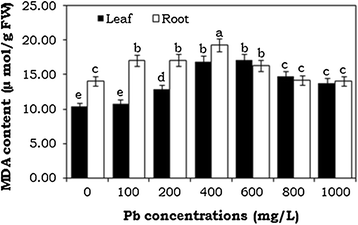

Supplement: Supplementary file 4 — Authors’ original file for figure 4 [file 40529_2014_9054_MOESM4_ESM.gif]

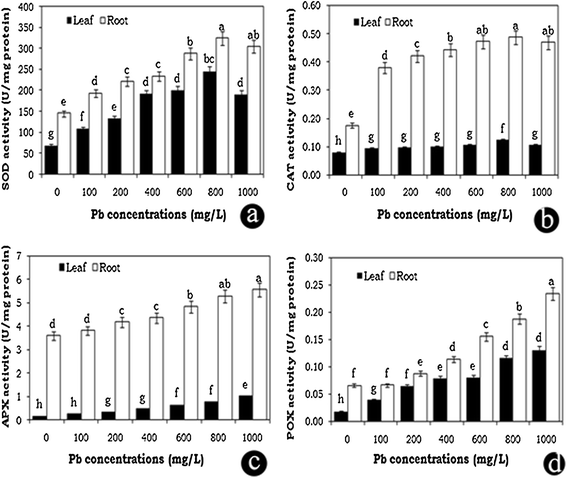

Supplement: Supplementary file 5 — Authors’ original file for figure 5 [file 40529_2014_9054_MOESM5_ESM.gif]
